# Metabolomic analysis of Agkistrodon haly venom poisoning mouse treatment by Jidesheng snake pill based on GC-MS

**DOI:** 10.3389/fphar.2024.1419609

**Published:** 2024-08-01

**Authors:** Jie Luo, Minkang Guo, Ke Xie, Ting-Li Han, Shanmu Ai

**Affiliations:** ^1^ Emergency Department, Chongqing University Central Hospital, Chongqing Emergency Medical Center, Chongqing, China; ^2^ Department of Critical Care Medicine, The First Affiliated Hospital of Chongqing Medical University, Chongqing, China; ^3^ The Chongqing Key Laboratory of Translational Medicine in Major Metabolic Diseases, Chongqing, China

**Keywords:** GC-MS, metabolomic, snakebite, Jidesheng, Agkistrodon haly venom

## Abstract

**Introduction:**

Snakebites are acute systemic toxic diseases caused by snake venom entering the body through wounds. Failure to use antivenom immediately and difficulty in obtaining antivenoms are frequently responsible for worsening disease. Traditional Chinese medicine is commonly used to supplement and replace antivenom in treating snakebites. The Jidesheng snake pill (JDS) is a widely used traditional Chinese medicine that has achieved good clinical therapeutic effects; however, its mechanism remains unclear. Therefore, metabolomics techniques were employed to explore the pathophysiological mechanisms of JDS treatment of Agkistrodon halys (Ah) snake venom-poisoned mice.

**Methods:**

The Ah group mouse model was established by intramuscular injection of Ah venom into the hind legs of the mice. The Ah venom + JDS group model was established using JDS after the affected area was treated with Ah venom. Hematoxylin and eosin (HE) staining was used to evaluate the severity of gastrocnemius injury. Quantitative polymerase chain reaction (qPCR) was utilized to detect the mRNA expression of vascular cell adhesion molecule-1 (VCAM-1), muscle-specific creatine kinase (CKM), thrombin antithrombin complex (TAT), and tumor necrosis factor-alpha (TNF-α). Gas chromatography-mass spectrometry (GC-MS) was performed with multivariate statistical analysis to provide new insights into the global metabolic profile of Ah venom-poisoned mice.

**Results:**

HE staining revealed increased red cell necrosis, local hemorrhage, and neutrophil infiltration in the Ah venom group than in the control group. Several compounds were identified, including lipids, amino acids, peptides, and organooxygen. Eighty differential metabolites were screened between the control group and the Ah venom group, and 24 were screened between the Ah venom and JDS groups. The mechanism of Ah venom poisoning in mice may involve aminoacyl-tRNA biosynthesis, various amino acid metabolism disorders, tricarboxylic acid circulation disorders, and abnormal fatty acid metabolism. JDS may reduce symptoms by affecting long-chain fatty acid and amino acid metabolism and promoting nicotinamide-nicotinamide metabolism.

**Conclusion:**

Our results suggest that metabolomics has huge prospects for elucidating the pathophysiology of Agkistrodon haly venom poisoning and therapeutic mechanisms of JDS.

## 1 Introduction

Snakebites affect 1.8–2.7 million people worldwide annually, resulting in an estimated 80,000–130,000 deaths (Longbottom et al., 2018). Chongqing is located in the southwestern part of China, with lush vegetation suitable for the growth and reproduction of snakes, including Agkistrodon halys (Ah), Protobothrops mucrosquamatus, Agkistrodon acutus, and Trimeresurus. The primary clinical symptoms of snakebites are progressive painful swelling and bleeding, which can be life-threatening in severe cases (Suhita et al., 2022). Hematotoxicity and cytotoxicity are the main manifestations of snakebites. Snakebites can have long-term physical after-effects, such as amputation, paralysis, disability, and mental health consequences (Ralph et al., 2022).

Antivenoms are currently the most effective treatment for snakebites; however, they have some limitations. First, antivenoms are expensive and difficult to obtain due to the complex production process required for their production and preservation (Chippaux, 2010). Second, antivenom has a poor effect on damaged organs (Trevett et al., 1995). Finally, some patients have severe allergic reactions to antivenom (Mahmoudi et al., 2021). In China, traditional Chinese medicine is used as a supplement and substitute for antivenoms (Huang and Hsieh, 2020; Ye et al., 2023). Jidesheng snake pill (JDS) is the most widely used and mainly contains Paris polyphylla Sm (qì yè yí zhīhuā), Toad Skin (chán chú pí), Centipede (wú gōng), and Euphorbia humifusa Willd (dì jíng cǎo). In the record of Chinese Pharmacopoeia 2020 edition, Polyphyllin was identified as the main component of JDS by HPLC, and its content was greater than 0.2 mg per tablet (0.4 g).

Its value is mentioned in ancient Chinese medicine books, such as Shennong Materia Medica, Materia Medica, and Compendium of Materia Medica. According to the modern medical theory, Paris polyphylla Sm. Affect heart muscle cells (Zeng et al., 2022). Toad skin is primarily used to treat tumors (Namba et al., 1989). Centipede and Euphorbia humifusa Willd have antibacterial effects (Ali et al., 2019; Lan et al., 2023), but the mechanism of action of JDS and its main components in snake bites has not been thoroughly elucidated.

Metabolomics is the quantitative analysis of low relative molecular mass metabolites of an organism or cell during a specific physiological period (Wu et al., 2023). Metabolomics technology can accurately reflect disease states through metabolites, allowing us to understand the development of the disease and provide possibilities for further treatment (DeBerardinis and Keshari, 2022). Simultaneously, changes in body function caused by therapeutic measures are reflected at the metabolomic level, which facilitates our interpretation of treatment results (Cutshaw et al., 2023). Recently, metabolomics techniques have been increasingly used to investigate the pathogenesis of diseases (Guo and Zhang, 2023). Metabolomics techniques have been applied to the early diagnosis and severity assessment of malignant tumors, cardiovascular and cerebrovascular diseases, poisoning, and other diseases (Sun et al., 2019; Arenas et al., 2023; Liu et al., 2024), as well as the development of new drug therapeutic targets (Qiu et al., 2023). Metabolomic analysis is a new method to study the mechanisms of action of traditional Chinese medicine and its derivatives (Wang et al., 2021). We tried to understand the changes in the body after snakebites using metabolomics techniques to provide the possibility for treating subsequent diseases.

In this study, we propose a metabolomics approach based on gas chromatography-mass spectrometry (GC-MS) to analyze metabolic changes in mice with Ah snake venom poisoning and evaluate the therapeutic targets and mechanisms of JDS for snake bites. We sought to provide a new perspective on snakebites and explore possible metabolic changes associated with snakebite disease.

## 2 Material and methods

### 2.1 Animals and drugs

Seventy-one 8–12-week male C57BL/6 wild-type (WT) mice (20 ± 2 g) were purchased from the Laboratory Animal Center of Chongqing Medical University and raised in an SPF animal room. The protocol was approved by the Institutional Animal Care and Use Committee of Chongqing Medical University (IACUC-CQMU-2023-0445). The room temperature was maintained at 24°C ± 2°C with humidity of 40%–45%. A 12-h light/dark cycle was set, and the mice were allowed free access to a standard diet and water. All subjects were given adaptive feeding for 2 weeks before the experiment.

JDS is manufactured by Essence Pharmaceutical Co., Ltd, jiangsu, China (Batch number 21220707; SFDA approval number Z32020048).

### 2.2 Compounds in JDS by UHPLC-MS/MS

The compounds in JDS were analyzed using the Vanquish™ ultrahigh-performance liquid chromatography (UHPLC) system (Thermo Fisher Scientific, Bremen, Germany). The chromatographic column used was the ACQUITY UPLC HSS-T3 (2.1 mm × 100 mm, 1.8 µm), with a column temperature of 35°C, flow rate of 0.3 mL/min, and total time of 20 min. The mobile phase comprised 0.1% aqueous formic acid solution (solvent A) and 0.1% Acetonitrile formate (solvent B). The gradient elution conditions are shown in Supplementary Table S1. Q-Exactive HFX mass spectrometer was combined with UHPLC system, and mass spectra were collected in positive and negative ion modes of ESI. (Supplementary Figure S1; Supplementary Figure S2; Supplementary Table S2).

### 2.3 Median lethal dose (LD_50_) calculation of ah venom

The median LD_50_ of Ah venom (Hunan Wolongtang Ascending Biotechnology Co., Ltd.) in mice fluctuated from 1 to 10 mg/kg in different studies. In this experiment, 36 mice were randomly selected and divided into six groups to determine the LD_50_ of Ah venom samples. Six groups of mice were injected with 0.1 mL snake venom solution of 1, 2, 4, 6, 8, and 10 mg/kg. Mouse death was recorded in each group within 7 days (Supplementary Table S3). The LD_50_ was calculated using probability unit regression in SPSS 23.0 (Sun et al., 2019). Simultaneously, a dose (2 mg/kg) with a survival rate of more than 90% and a local response was selected as the test dose (Supplementary Table S4).

### 2.4 Establishment and grouping of snakebite model

Twenty-one mice were divided into the control (n = 3), Ah venom (n = 9), and Ah venom + JDS (n = 9) groups. Snake venom solution (1 mg/mL) was prepared with phosphate buffered saline (PBS)solution and lyophilized powder of snake venom. The mice were anesthetized with an intraperitoneal injection of 1% phenobarbital sodium (40 mg/kg), and the skin was prepared on the right hind leg. In the Ah venom and Ah venom + JDS groups, 0.04 mL snake venom solution was injected into the gastrocnemius muscle to establish the disease model of snake bite. The mice in the Ah venom + JDS group were given an external application of JDS 0.4 g + PBS (3 mL) to the right hind leg twice daily.

According to the time of specimen collection after modeling, the Ah venom group was divided into 4 h Ah venom (n = 3), 24 h Ah venom (n = 3), and 7 days Ah venom (n = 3) groups. The Ah venom + JDS group was divided into 4 h Ah venom + JDS (n = 3), 24 h Ah venom + JDS (n = 3), and 7 days Ah venom + JDS (n = 3) groups. None of the mice died before specimen collection.

### 2.5 Sample collection and processing

Specimens were collected at 4 h, 24 h, and 7 days after establishing the snakebite mouse model. Mice were fully anesthetized with an intraperitoneal injection of 1% sodium phenobarbital (40 mg/kg). The beards were trimmed, the eyeballs were removed with curved forceps, and blood was collected using a sterile Eppendorf (EP) tube rinsed with 0.1 mL heparin. The EP tube was mixed up and down to obtain a full anticoagulant. The right hind leg was fully exposed. The gastrocnemius muscle was removed, rinsed with sterile PBS), and fixed in 4% paraformaldehyde for morphological examination. The remainder was rinsed with sterile PBS and transferred to a dry EP tube for examination.

### 2.6 Blood routine test

Blood samples were analyzed within 0.5 h using an automatic animal blood analyzer XT-2000i (SYSMEX Co., LTD., Japan). White blood cells, platelets, and red blood cells from the two mice groups were compared.

### 2.7 Hematoxylin and eosin (HE) staining

Fresh tissue from the mouse lungs was fixed for 24 h with 10% paraformaldehyde and sectioned after routine dehydration and paraffin embedding. Pathological changes in the lungs were observed under a light microscope after HE staining.

### 2.8 ELISA kits

The muscle tissue was washed with precooled PBS to remove residual blood and cut into pieces after weighing. The scission tissue was mixed with a corresponding volume of PBS and recorded. Protease inhibitors were added to a glass homogenizer after adding PBS and ground thoroughly on ice. The final homogenate was centrifuged at 5,000 × *g* for 5 min, and the supernatant was used for detection. CKM, TAT, and VCAM-1 levels were measured using ELISA kits.

### 2.9 RNA isolation and quantitative PCR (qPCR)

Total RNA was extracted from the muscle tissue using TRIzol reagent and DNase I digestion, according to the manufacturer’s instructions (Lei and Sun, 2018). cDNA was generated from the total RNA extracted from the tissues using a reverse transcription reaction kit (TAKARA, Japan). The cDNA was used as a template for subsequent qPCR assays. The primers used in this study are listed in Supplementary Table S5.

### 2.10 Metabolites extraction

The muscle tissue (20 mg) was mixed with 500 µL of cold methanol-water (50% v/v) in a ball mill for 10 min. The samples were prepared as previously described (Guo and Zhang, 2023). The metabolites were extracted using a sequence of the following solvents: 300 µL tridecanoic acid (2.5 mg/mL) in ethyl acetate: ethyl alcohol (1:1); 200 µL methanol; 200 µL methanol: H_2_O (3:1); 200 µL dichloromethane: methanol (1:1). The liquid was mixed with the samples and the supernatant was collected by centrifugation at 1,000 *g* for 5 min. Briefly, 20 µL of liquid was mixed from each sample to obtain a quality control (QC) sample. All samples were dried using an MTN-2800D concentrator. The metabolites in the samples were silylated and methoxylated, as shown below. Briefly, 167 µL of methanol and 34 µL of pyridine were added as the methyl donor and catalyst, respectively. Subsequently, 200 µL of sodium hydroxide (1 M) was added. The reaction was initiated by adding 20 µL methyl chlorate and 30-s rotation. Methyl chlorate (20 µL) was then added, and the mixture was rotated for another 30 s. Briefly, 400 µL chloroform and 400 µL sodium bicarbonate (50 mM) were added and rotated for 10 s, the lower chloroform phase was separated, and anhydrous sodium sulfate was added to remove excess water. Finally, the derived metabolites were isolated from the reaction mixture. The sample mixture (100 µL) was transferred to a vial before the GC-MS analysis.

### 2.11 GC/MS analysis

The metabolites were analyzed using an Agilent 6890 N/5,973 N series GC-MS system. Compounds were identified according to two criteria: >85% spectral match with our library and within 1 min of the corresponding chromatographic retention time. The relative abundance of metabolites was extracted using in-house MassOmics software, and the peak height of the highest reference ion mass was calculated. The temperature was maintained at 85°C for 3 min and then raised to 280°C at a rate of 10°C/min. The samples were rapidly injected in the split mode at 260°C. Mass spectra were obtained in full scan mode using repeated scans from 60 to 600 m/z. The injection volume used was 1 µL. The compounds were statistically analyzed, deconvolved, and identified using an automated mass spectrometry deconvolution and identification system (AMDIS) based on a self-developed methyl chloroformate derivatization mass spectrometry library. Quick peak-view technology was used to promote fragment ion analysis using a peak-matching algorithm. The data were peak-detected, and noise was reduced, leaving only true analytical peaks for further processing.

### 2.12 Data processing and analysis

The fragment ion analysis process was enhanced by implementing quick peak view technology using a peak-matching algorithm. Following peak detection, the data were reduced to noise to ensure that only the genuine analytical peaks were subjected to further processing. The Rt-m/z data pairs were used as identifiers, and this process was repeated for each analysis. In a table, the data were sorted to align the correct peak intensity data for each Rt-m/z pair. MarkView software was used to extract, pre-process, and normalize all ion features. The pre-processed data matrix was imported into MetaboAnalyst (https://www.metaboanalyst.ca/) for multivariate statistical and pathway analyses.

### 2.13 Statistical analysis

Statistical analyses were performed using MetaboAnalyst (https://www.metaboanalyst.ca/). All quantitative experiments were performed in triplicate. Metabolites significantly differed when | logFC | > 0.5 and *p* < 0.05. GraphPad Prism 8.0 was used for plotting. Data are expressed as mean ± standard deviation (SD). Student’s t-test was used for pairwise comparisons. A *p*-value <0.05 was statistically significant.

## 3 Results

### 3.1 Histological analysis of muscle tissue

**FIGURE 1:**
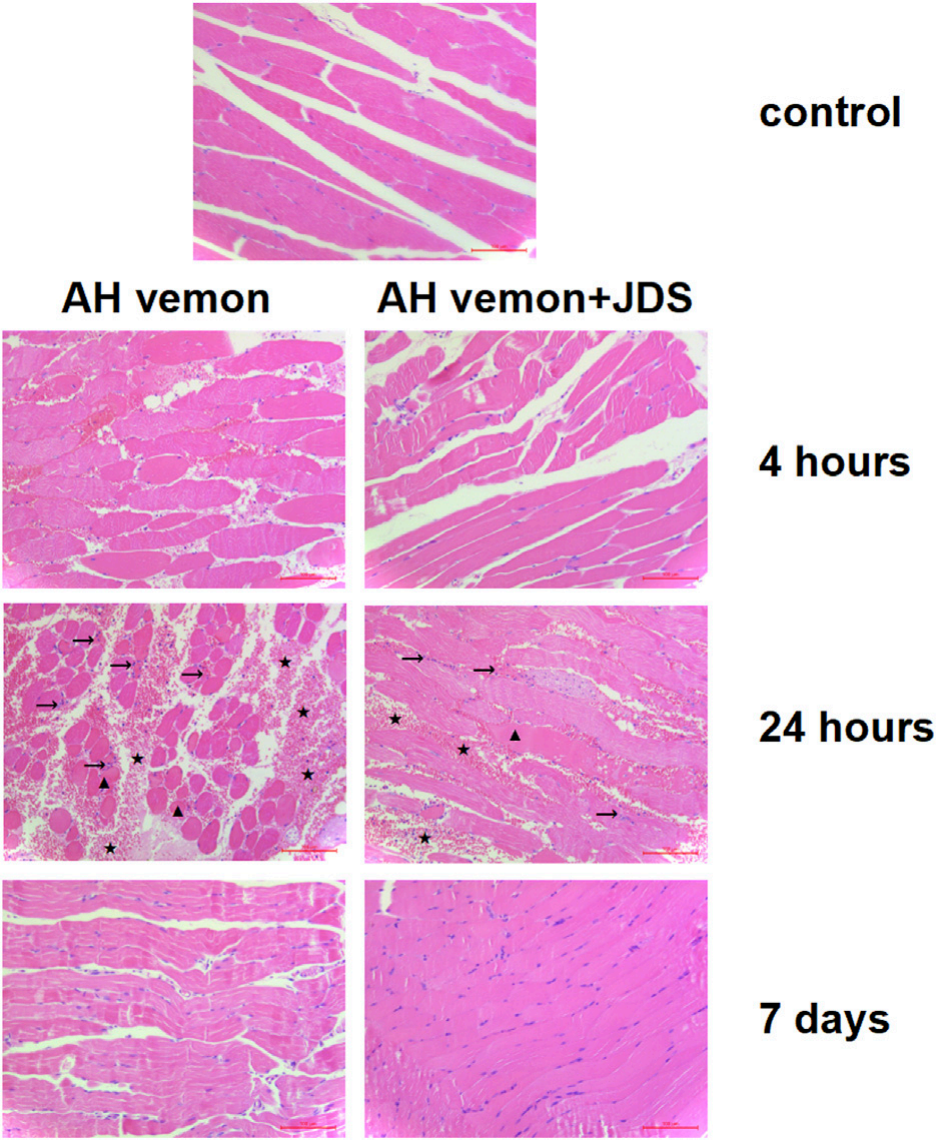
Histological image of muscle tissue samples. The mice showed muscle cell necrosis (▲), local hemorrhage (★), and neutrophil infiltration (→) as the main manifestations after modeling. The local pathological changes began to appear at 4 h after modeling, which was most obvious at 24 h and significantly improved after 7 days.

The mice showed muscle cell necrosis, local hemorrhage, and neutrophil infiltration as the main manifestations after modeling compared, with the control group. Local pathological changes began to appear at 4 h after modeling, which was most obvious at 24 h and significantly improved after 7 days. The Ah venom + JDS group showed a dynamic change trend similar to that of the Ah venom group but with lighter pathological changes at each time point than the venom group (Figure 1).

### 3.2 Detection of red blood cells, white blood cells, and platelets in mice

Compared with the control group, the leukocyte counts of mice showed a downward trend in the Ah venom and Ah venom + JDS groups; the change was most obvious at 24 h, and the trend was improved at 7 days. At different time points, the decreased degree of leukocyte count was lower in the Ah venom + JDS group than in the Ah venom group. The 4 h Ah venom + JDS, 24 h Ah venom + JDS, 4 h Ah venom, and 24 h Ah venom groups showed significant differences (*p* < 0.01).

Compared with the control group, the erythrocyte counts of the Ah venom and Ah venom + JDS groups showed a downward trend, and the change was most obvious at 24 h and improved at 7 days. After forming the disease model, erythrocytes showed a progressive downward trend without improvement observed during the observation period. The Ah venom + JDS and Ah venom groups did not differ significantly at different time points (*p* > 0.05).

**FIGURE 2:**
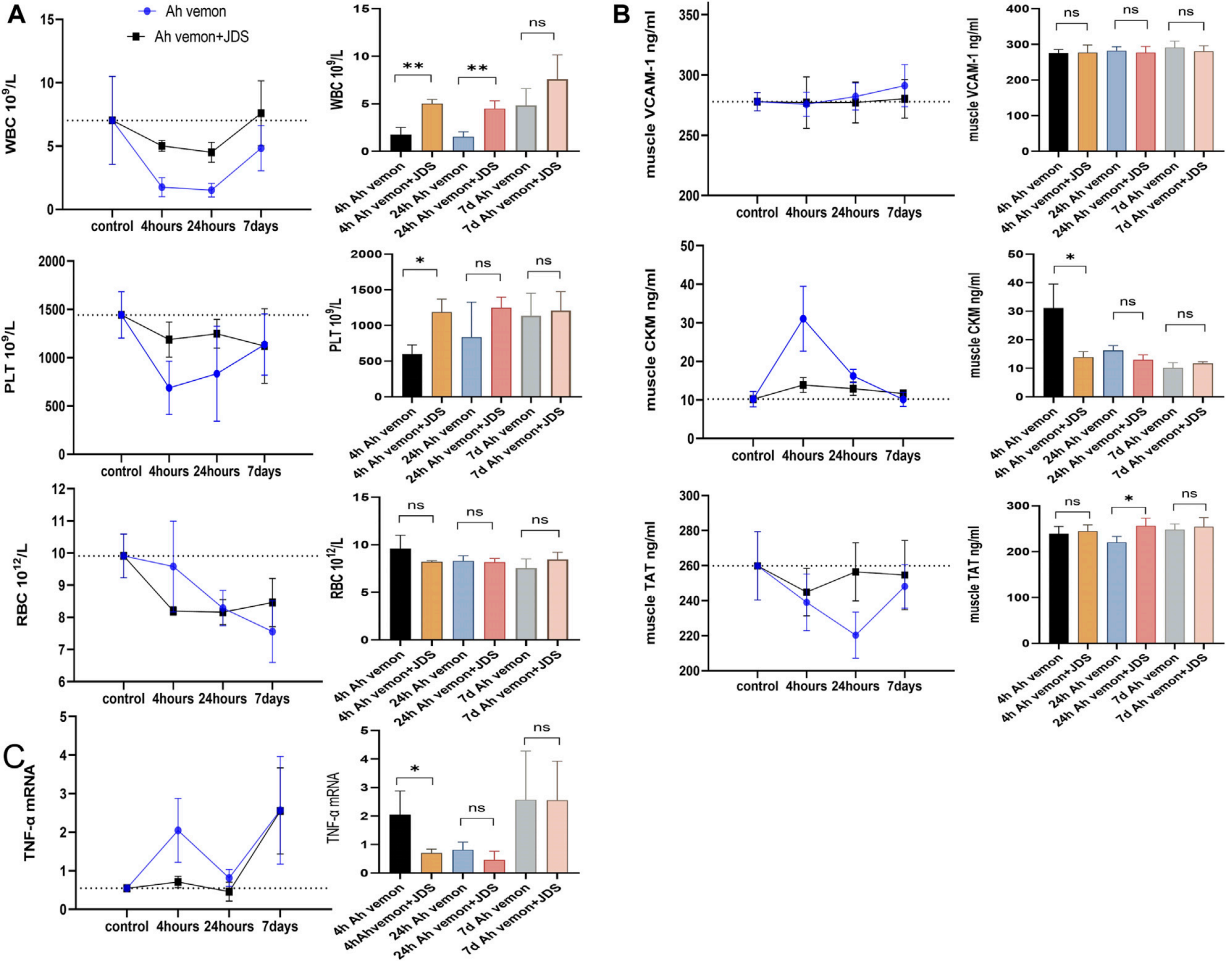
Blood routine examination, ELISA, qPCR for mice tissues. **(A)** The levels of WBC, RBC and PLT in mouse. **(B)** The Levels of vcam-1, ckm, and TAT in mouse muscle tissue homogenate. **(C)** The expression of TNF-a mRNA in mouse muscle tissue homogenate. WBC, white blood cell; PLT, blood platelet; RBC, Red blood cell. VCAM-1, vascular cell adhesion molecule-1; TAT, thrombin antithrombin complex; CKM, muscle-specific creatine kinase; TNF-a, Tumour necrosis factor alpha. (*p < 0.05, **p < 0.01, and ns mean *p* > 0.05).

Compared with the control group, the platelet counts of mice in the Ah venom and Ah venom + JDS groups exhibited a decreasing trend, and the change was most obvious at 4 h and gradually improved at 24 h and 7 days. The decrease in platelet count was lower in the Ah venom + JDS group than that in the Ah venom group at different time points. The Ah venom + JDS and 4 h Ah venom groups differed significantly (*p* < 0.05, Figure 2A).

### 3.3 Detection of enzymes in mouse muscle homogenates using ELISA

VCAM-1 is rapidly activated by vascular endothelial cells in the inflammatory state and functions as a cell adhesion molecule, mediating the arrival of leukocytes, monocytes, and neutrophils at the site of inflammation (Wei et al., 2023). Compared with the control group, VCAM-1 expression in the muscle homogenate of the Ah venom and Ah venom + JDS groups revealed an increasing trend, and the change was gradually obvious with time at 4 h, 24 h, and 7 days. VCAM-1 expression in the muscle homogenate of the Ah venom + JDS group was lower than that of the Ah venom group in the disease group at different time points, without statistical significance (*p* > 0.05).

CKM is an enzyme expressed in various tissues, and its serum concentration is used as a biomarker of muscle injury (Fernández-Torres et al., 2021). Compared with the control group, CKM expression in the muscle homogenate of the Ah venom and Ah venom + JDS groups showed an increasing trend, and the change was most obvious at 4 h, while it had a gradually decreasing trend at 24 h and 7 days. CKM expression was lower in the rat muscle homogenate of the Ah venom + JDS group than in the Ah venom group at different time points. The difference between the Ah venom + JDS and 4 h Ah venom groups was statistically significant (*p* < 0.05).

TAT has been linked to muscle ischemia/reperfusion injury and is a biomarker of an organism’s hypercoagulable state (Zhou et al., 2024). Compared with the control group, TAT expression in the muscle homogenate of the Ah venom and venom + JDS groups exhibited a downward trend, and the change was most obvious in the Ah venom group at 24 h and recovered at 7 days. The Ah venom + JDS group change was most obvious at 4 and 24 h, and 7 days showed a gradual increase. CKM expression was lower in the rat muscle homogenate of the Ah venom + JDS group than that in the Ah venom group at different time points. The difference between the 24 h Ah venom + JDS and 24 h Ah venom groups was significant (*p* < 0.05, Figure 2B).

### 3.4 Detection of TNF-α mRNA in mouse muscle homogenates using qPCR

Compared with the control, Ah venom, and Ah venom + JDS groups, TNF-α mRNA expression in mouse muscle homogenates showed an upward trend. The increased rate of TNF-α mRNA was lower in the muscle homogenate of the Ah venom + JDS group than that in the Ah venom group at different time points. At 4 h, TNF-α expression was significantly increased in the Ah venom group compared with the Ah venom + JDS group, and the difference was statistically significant (*p* < 0.05, Figure 2C).

### 3.5 Metabolomic analysis of mouse muscle

#### 3.5.1 Data analysis before processing

**FIGURE 3:**
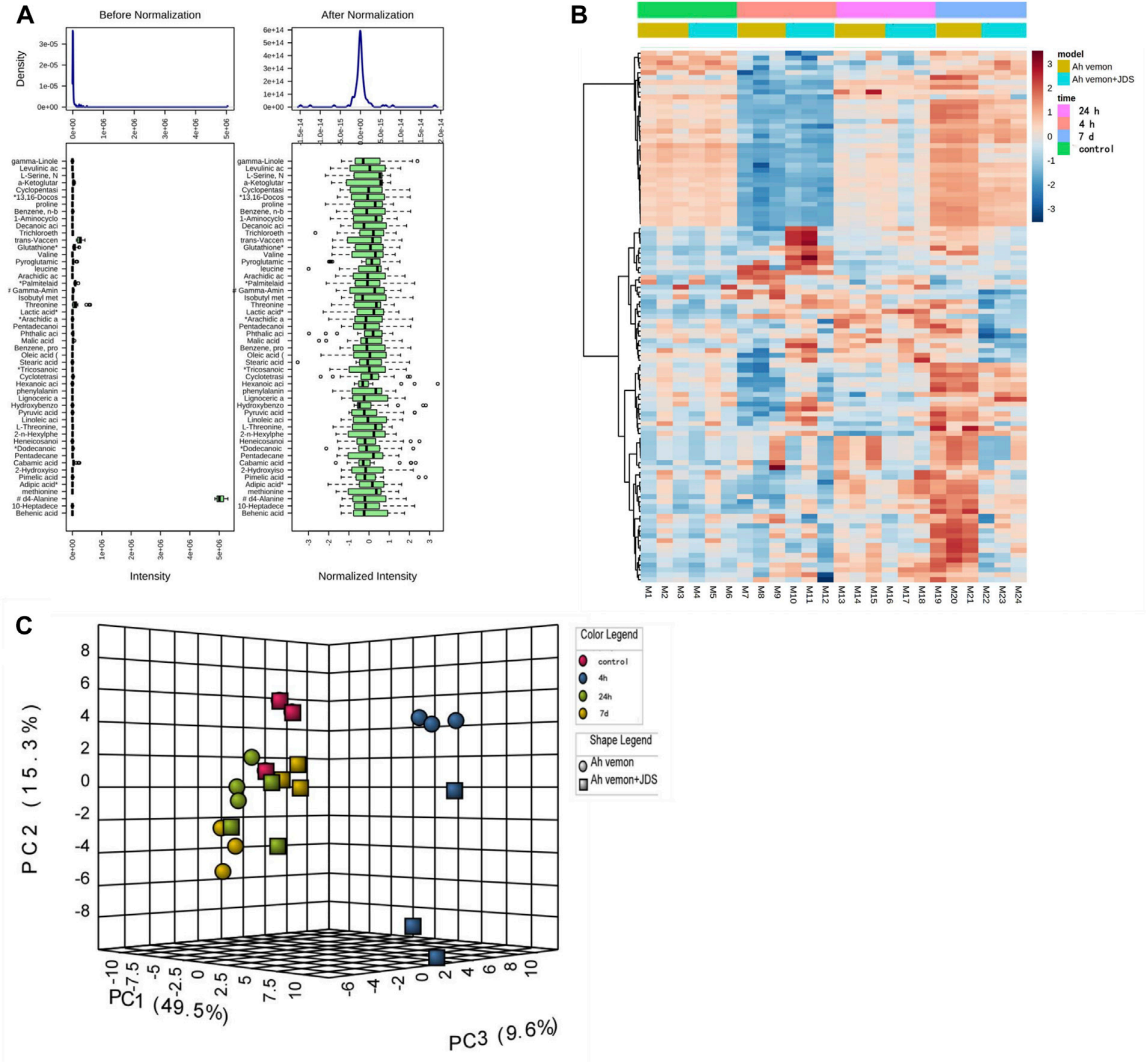
Heatmap of identified metabolites. **(A)** Box plots and kernel density plots before and after normalization. **(B)** The visual heat map. **(C)** Principal Component Analysis (PCA).

Before the statistical analysis, the missing value was used to retain variables with non-missing values greater than 80%, and the remaining “0” value was assigned the average value. Some unmatched metabolites were identified by comparing physicochemical properties and/or database similarity of reference substances, and metabolites with a >85% matching degree were used. Sample weight normalization and logarithmic transformation of the data were performed (Figure 3A).

#### 3.5.2 Analysis of metabolites in mouse muscle tissue

**FIGURE 4:**
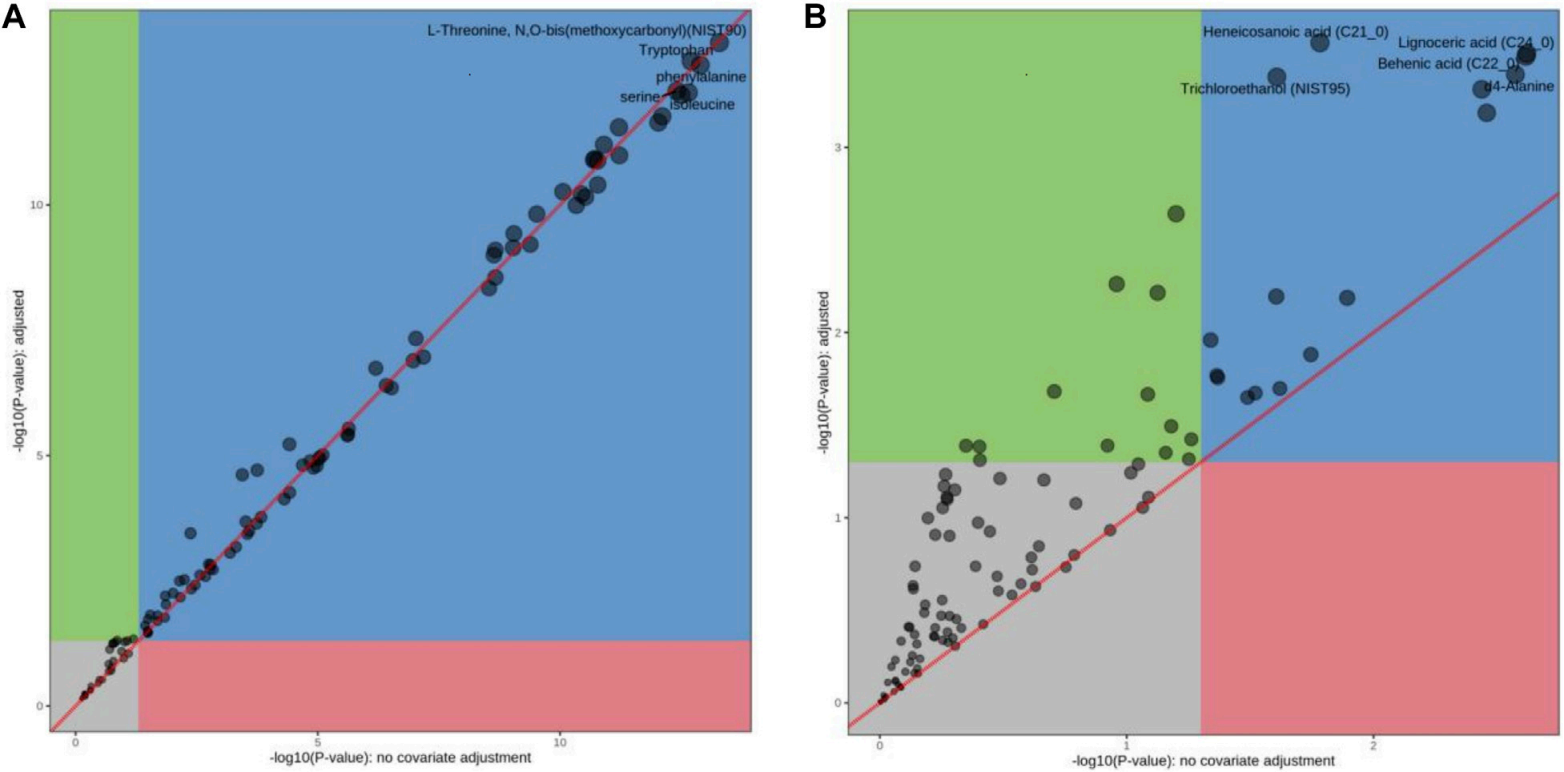
The differences in the metabolites between the two sequences were screened using limma analysis. **(A)** limma analysis of Ah vemon group vs control group. **(B)** limma analysis of Ah vemon + JDS group vs Ah vemon group.

Principal component analysis (PCA) is a statistical analysis tool that visualizes the differences between metabolite levels under the influence of different factors (Debik et al., 2022). The heatmap reveals the standard concentration of each metabolite in each sample on a false-color scale. The samples and metabolites were organized according to the corresponding hierarchical clustering tree (Pietrafesa et al., 2023). In this study, a heatmap (Figure 3B) and PCA (Figure 3C) displayed a good clustering effect. The differences in the metabolites between the two sequences were screened using limma analysis. When time was used as the independent variable, 80 metabolites were differentially expressed in the 4 h, 24 h, and 7 days groups than in the control group (either | logFC | > 0.5, *p* < 0.05, Supplementary Table S6; Figure 4A). Among them, 24 metabolites were upregulated, whereas 56 were downregulated. The top five upregulated distributions were succinic acid, citraconic acid, glyoxylic acid, malonic acid, and tricosanoic acid. The top five downregulated metabolites were L-threonine, tryptophan, phenylalanine, serine, and isoleucine. When the before and after treatments were the independent variables, 29 metabolites were differentially expressed in the Ah venom + JDS group than in the Ah venom groups at different time points (either | logFC | > 0.5, *p* < 0.05). (Supplementary Table S7; Figure 4B). Among them, adipic acid and tridecanoic acid were upregulated, whereas the other 27 metabolites were downregulated. The top five downgrades of the 29 metabolites were heneicosanoic acid, lignoceric acid, behenic acid, D4-alanine, and trichloroethane.

### 3.6 Metabolic pathway analysis

**FIGURE 5:**
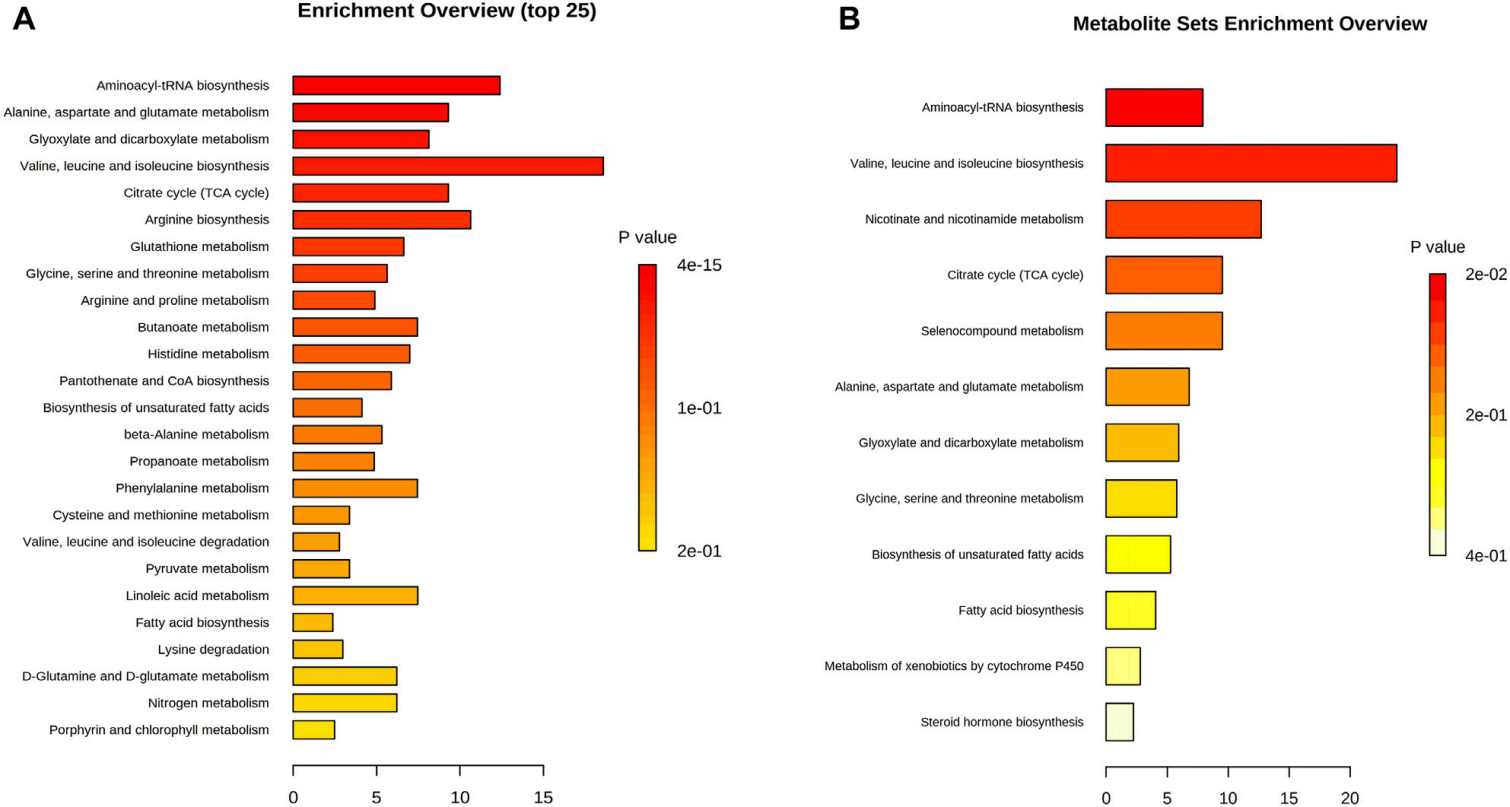
Metabolism pathway analysis. **(A)** Ah vemon groups vs control group. **(B)** Ah vemon + JDS groups vs Ah vemon groups.

Topology and enrichment analyses were performed using metaboanalyst 5.0 (https://www.metaboanalyst.ca/) to screen for metabolic pathways. 1) Compared with the control group, 39 metabolic pathways were enriched in the Ah venom groups. Among these, 17 different metabolic pathways were screened (*p* < 0.05, Figure 5A). 2) Compared with Ah venom groups, 12 metabolic pathways were enriched in the Ah venom + JDS groups. Among these, six metabolic pathways were screened (*p* < 0.05, Figure 5B).

## 4 Discussion

Venomous snake bites are an acute health problem worldwide. In 2009 and 2017, the General Assembly of the World Health Organization (WHO) adopted relevant documents on treating snakebites, requiring more attention and increasing research investment (Minghui et al., 2019). Although antivenin is the most effective treatment for venomous snake bites, it cannot effectively treat pathophysiological changes in the target organs after poisoning. Currently, traditional Chinese medicine (TCM), represented by JDS, remains the key medicine for treating venomous snake bites in China. This study examined the effectiveness of JDS as a therapeutic drug for local envenomation in mice. This study utilized metabolomics to compare pathophysiological processes in mice before and after treatment to determine the progression of AH venomous snake bites from local to systemic. The metabolomic mechanisms of Ah snakebites and JDS in treating snake bites were explored to provide new ideas for treating snake bites.

In this study, the median LD_50_ was calculated as 4.98 mg/kg, and a dose of 2 mg/kg (survival rate >92%) that could produce toxic reactions and ensure the survival of model mice was selected. Compared with the control group, HE staining disclosed that the main manifestations of the Ah venom group mice were local inflammatory infiltration, myofibrocyte destruction, and bleeding, and the pathological changes at 24 h were more obvious than those at 4 h and 7 days. Existing studies have found pathological changes in the gastrocnemius muscle 24, 48, and 72 h after snakebites, which is consistent with our study and indicates that the muscle begins to repair after 48 h (Campos et al., 2018). By comparing the Ah venom and Ah venom + JDS groups, we found that the pathological changes in the Ah venom + JDS group were lighter than those in the Ah venom group at various time points. These results indicate that JDS could change the muscle injury caused by snake venom based on histopathology. As a product of skeletal muscle destruction, CKM can reflect the degree of muscle necrosis caused by snakebites (Alsolaiss et al., 2022). CKM levels in muscle homogenates increased significantly after the establishment of the mouse poisoning model. CKM expression was lower in the muscle homogenate of the Ah venom + JDS group than that in the Ah venom group at different time points. In this study, muscle damage occurred after modeling the snake venom poisoning model in mice, and JDS has a certain effect in inhibiting muscle damage in injured mouse models**.**

In the local inflammatory response, white blood cells in mice exhibited a downward trend after modeling. Studies have now reported the increase and decrease in white blood cells in a mouse model of local snake venom poisoning, which is considered to be linked to the type of snake venom. However, the increase or decrease is considered to be caused by local inflammation and stress response (Sebastin Santhosh et al., 2013). We found that the white blood cell count was significantly higher in the Ah venom + JDS group than in the Ah venom group at 24 and 48 h (*p* < 0.01). TNF-α mRNA expression presented an increasing trend in the Ah venom group mice, and its increasing process had an increasing trend 4 h after modeling, falling 24 h after modeling, and increasing 7 days after modeling. Combined with existing studies, TNF-α mRNA as a pro-inflammatory factor was significantly increased in the 4 h mouse model due to oxidative stress and acute inflammation (Gabrili et al., 2023), and the increase again at 7 days may be correlated with the activation and proliferation of satellite cells in the regeneration stage of skeletal muscle (Sanchez-Castro et al., 2021). Our study found that TNF-α mRNA expression was lower in the Ah venom + JDS group at 4 and 24 h than in the Ah venom group, without difference at 7 days. Previous studies have found that human microvascular endothelial cells (HMEC) stimulated by complement bypass activation products mediated by cobra venom factors can upregulate the expression of adhesion molecules (ICAM-1, VCAM-1, and E-selectin) (Acunha et al., 2021; Mota et al., 2021), consistent with our findings. In this study, the Ah venom + JDS and Ah venom groups exhibited downward trends. JDS can alleviate the inflammatory effect and improve the local inflammation-related indicators of snakebite model mice.

Other studies have demonstrated that procoagulant toxins in snake venom promote consumptive coagulation disorders, leading to the consumption of clotting substrates, and that snake venom can also directly promote intravascular hemolysis and cause thrombocytopenia (Isbister, 2010; Maduwage and Isbister, 2014). In this study, platelet and TAT levels in mice decreased to varying degrees after modeling, and the decrease was smaller in the Ah venom + JDS group than in the Ah venom group at all three-time points. Platelet changes differed significantly between the 4 h Ah venom + JD and 4 h Ah venom groups (*p* < 0.05). TAT levels were significantly different between the 24 h Ah venom + JDS group and 24 h Ah venom group (*p* < 0.05). The results showed that platelets, prothrombin complex, and other clotting substrates were consumed after establishing a mouse model of local envenomation, and JDS improved the clotting function of the snakebite model mice.

Metabolomics technology explores the dynamic process of toxicity onset, development, and metabolism of toxicants *in vivo* via comprehensive qualitative and quantitative analyses of compounds in dynamic and static states (Baidoo et al., 2012a). Simultaneously, when treatment measures are changed, early, timely, and subtle changes at the metabolome level have become important markers and developed into an effective means of real-time monitoring, evaluation, and guidance for individualized treatment (Baidoo et al., 2012b). As the target organ was directly injured by snake venom in our study, the gastrocnemius muscle can react earlier to the effect of snake venom on the body, and the same trend was observed in the above studies. In our study, the gastrocnemius muscle of the affected side of mice was used as the research object, and the concentration of the metabolites was determined using GC-MS. Eighty differential metabolites were screened before and after modeling; 61 peaked in the early stage of disease (4 h), while 53 returned to near-normal levels 7 days later (|logFC| < 1). The upregulated metabolites were mainly glucose and fatty acid metabolites, such as succinic acid, cyclotonic acid, glyoxylic acid, malonic acid, and tridecanoic acid, while the downregulated metabolites were mainly amino acids and their metabolites, such as L-threonine, tryptophan, phenylalanine, serine, and isoleucine. Among the 17 metabolic pathways, five were the most common: 1) aminoacyl-tRNA biosynthesis; 2) alanine, aspartate, and glutamate metabolism; 3) glyoxylate and dicarboxylate metabolism; 4) valine, leucine, and isoleucine biosynthesis; 5) the citrate cycle (TCA) (*p* < 0.05, FDR <0.01). Our study found that various amino acid and metabolite levels were significantly reduced, and various amino acid metabolic pathways were blocked. However, snake venom contains many proteolytic enzymes, mainly serine hydrolases, leading to rapid amino acid consumption after poisoning (Mickiewicz et al., 2013), which can affect protein synthesis by blocking acyl-tRNA biosynthesis (Sissler, 2021). The upregulated product of citric acid is also the citric acid metabolite after heating, suggesting that the main intermediate metabolites, such as succinic acid and cyclotonic acid in the tricarboxylic acid cycle are upregulated, and citric acid and malic acid are reduced. These results indicated that the mitochondrial energy supply in the gastrocnemius muscle of mice with the TCA cycle was disturbed after the snake venom poisoning model was constructed. Simultaneously, metabolic intermediate accumulation, such as succinic acid, reduces ATP production and acts as a pro-inflammatory mediator to induce local inflammation (Udvardy et al., 2020). Among other major upregulation products, tridecanoic acid is a long-chain fatty acid present in cell membranes (Stifel et al., 2022), and its increased detection should be considered because of cell structure destruction caused by snake venom phospholipase and degradation of triglycerides in cell membranes (Ní et al., 2012). Long-chain fatty acids can also promote the occurrence and development of local inflammation and aggravate inflammatory response (Yanagisawa et al., 2008).

In this study, the metabolite changes in the Ah venom and Ah venom + JDS groups were compared at 4 h, 24 h, and 7 days after modeling, and 23 metabolites were differentially expressed, among which 2 (adipic acid and tridecanoic acid) were upregulated, and 21 were downregulated. The top five compounds identified were heneicosanoic acid, lignoceric acid, behenic acid, d4-alanine, and d4-alanine. Twelve metabolic pathways were involved; among these, six metabolic pathways were screened (*p* < 0.05). The levels of multi-ultra-long-chain fatty acids (C ≥ 20) were significantly increased in these changes, whereas the changes were reduced in the treatment group. For example, lignoceric acid, an important component of phosphatidylcholine (Uhrig et al., 1997), is involved in constructing cell membranes in snake venom under the action of phospholipase A2. The reduction in lignoceric acid after treatment can be attributed to the reduction in phosphatidylcholine degradation. Concurrently, ultra-long-chain fatty acid metabolism requires the participation of peroxidase, and the dysfunction of peroxidase bodies leads to further accumulation of ultra-long-chain fatty acids (Matsumori et al., 2013). Nicotinamide is a metabolite of vitamin B3 that has anti-inflammatory effects (Patil et al., 2023). Nicotinamide adenine dinucleotide phosphate (NADH) can be produced via the nicotinamide metabolic pathway, and oxidative phosphorylation of NADH can produce a large amount of ATP to maintain life activities (Luo et al., 2023).

## 5 Conclusion

The mechanism of Ah venom poisoning in mice may involve aminoacyl-tRNA biosynthesis, various amino acid metabolism disorders, tricarboxylic acid circulation disorders, and abnormal fatty acid metabolism. JDS can reduce symptoms by affecting the metabolism of long-chain fatty acids and amino acids, promoting nicotinamide-nicotinamide metabolism.

## Data Availability

The original contributions presented in the study are included in the article/Supplementary Material, further inquiries can be directed to the corresponding author.

## References

[B1] Acunha T. Nardini V. Faccioli L. H. (2021). A lipidomics approach reveals new insights into *Crotalus durissus* terrificus and Bothrops moojeni snake venoms. Arch. Toxicol. 95 (1), 345–353. 10.1007/s00204-020-02896-y 32880718

[B2] Ali S. M. Khan N. A. Sagathevan K. Anwar A. Siddiqui R. (2019). Biologically active metabolite(s) from haemolymph of red-headed centipede Scolopendra subspinipes possess broad spectrum antibacterial activity. Amb. Express 9 (1), 95. Published 2019 Jun 28. 10.1186/s13568-019-0816-3 31254123 PMC6598926

[B3] Alsolaiss J. Evans C. A. Oluoch G. O. Casewell N. R. Harrison R. A. (2022). Profiling the murine acute phase and inflammatory responses to african snake venom: an approach to inform acute snakebite pathology. Toxins (Basel). 14 (4), 229. Published 2022 Mar 22. 10.3390/toxins14040229 35448838 PMC9028489

[B4] Arenas M. Fargas-Saladié M. Moreno-Solé M. Moyano-Femenia L. Jiménez-Franco A. Canela-Capdevila M. (2023). Metabolomics and triple-negative breast cancer: a systematic review. Heliyon 10 (1), e23628. Published 2023 Dec 13. 10.1016/j.heliyon.2023.e23628 38187259 PMC10770474

[B5] Baidoo E. E. Benke P. I. Keasling J. D. (2012a). Mass spectrometry-based microbial metabolomics. Methods Mol. Biol. 881, 215–278. 10.1007/978-1-61779-827-6_9 22639216

[B6] Baidoo E. E. Benke P. I. Keasling J. D. (2012b). Mass spectrometry-based microbial metabolomics. Methods Mol. Biol. 881, 215–278. 10.1007/978-1-61779-827-6_9 22639216

[B7] Campos G. R. S. de Moura K. M. B. Barbosa A. M. Zamuner L. F. Nadur-Andrade N. Dale C. S. (2018). Light emitting diode (LED) therapy reduces local pathological changes induced by *Bothrops asper* snake venom. Toxicon 152, 95–102. 10.1016/j.toxicon.2018.07.029 30081063

[B8] Chippaux J. P. (2010). Guidelines for the production, control and regulation of snake antivenom immunoglobulins. Biol. Aujourdhui 204 (1), 87–91. 10.1051/jbio/2009043 20950580

[B9] Cutshaw G. Uthaman S. Hassan N. Kothadiya S. Wen X. Bardhan R. (2023). The emerging role of Raman spectroscopy as an omics approach for metabolic profiling and biomarker detection toward precision medicine. Chem. Rev. 123 (13), 8297–8346. 10.1021/acs.chemrev.2c00897 37318957 PMC10626597

[B10] DeBerardinis R. J. Keshari K. R. (2022). Metabolic analysis as a driver for discovery, diagnosis, and therapy. Cell. 185 (15), 2678–2689. 10.1016/j.cell.2022.06.029 35839759 PMC9469798

[B11] Debik J. Sangermani M. Wang F. Madssen T. S. Giskeødegård G. F. (2022). Multivariate analysis of NMR-based metabolomic data. NMR Biomed. 35 (2), e4638. 10.1002/nbm.4638 34738674

[B12] Fernández-Torres J. Martínez-Nava G. A. Zamudio-Cuevas Y. Barbier O. C. Narváez-Morales J. Martínez-Flores K. (2021). Ancestral contribution of the muscle-specific creatine kinase (CKM) polymorphism rs4884 in the knee osteoarthritis risk: a preliminary study. Clin. Rheumatol. 40 (1), 279–285. 10.1007/s10067-020-05238-6 32557253

[B13] Gabrili J. J. M. Pidde G. Magnoli F. C. Marques-Porto R. Villas-Boas I. M. Squaiella-Baptistão C. C. (2023). New insights into immunopathology associated to Bothrops lanceolatus snake envenomation: focus on PLA2 toxin. Int. J. Mol. Sci. 24 (12), 9931. Published 2023 Jun 9. 10.3390/ijms24129931 37373079 PMC10298673

[B14] Guo M. Zhang J. (2023). Metabolomic analysis of bone-derived exosomes in osteonecrosis of the femoral head based on UPLC-MS/MS. Metabolomics 19 (4), 34. Published 2023 Apr 1. 10.1007/s11306-023-01986-z 37002424

[B15] Huang T. I. Hsieh C. L. (2020). Effect of traditional Chinese medicine on long-term outcomes of snakebite in taiwan. Toxins (Basel) 12 (2), 132. Published 2020 Feb 20. 10.3390/toxins12020132 32093388 PMC7076781

[B16] Isbister G. K. (2010). Snakebite doesn't cause disseminated intravascular coagulation: coagulopathy and thrombotic microangiopathy in snake envenoming. Semin. Thromb. Hemost. 36 (4), 444–451. 10.1055/s-0030-1254053 20614396

[B17] Lan T. Chen H. F. Zheng F. Huang H. Wu Q. Fan X. Y. (2023). Cinobufacini retards progression of pancreatic ductal adenocarcinoma through targeting YEATS2/TAK1/NF-κB axis. Phytomedicine 109, 154564. 10.1016/j.phymed.2022.154564 36610152

[B18] Lei C. Sun X. (2018). Comparing lethal dose ratios using probit regression with arbitrary slopes. BMC Pharmacol. Toxicol. 19 (1), 61. Published 2018 Oct 5. 10.1186/s40360-018-0250-1 30290834 PMC6173863

[B19] Liu J. Wu Y. Zhu Y. Yu C. Zhang Y. Luo T. (2024). A new insight into mechanism of colchicine poisoning based on untargeted metabolomics. Phytomedicine 122, 155122. 10.1016/j.phymed.2023.155122 37863002

[B20] Longbottom J. Shearer F. M. Devine M. Alcoba G. Chappuis F. Weiss D. J. (2018). Vulnerability to snakebite envenoming: a global mapping of hotspots. Lancet 392 (10148), 673–684. 10.1016/S0140-6736(18)31224-8 30017551 PMC6115328

[B21] Luo Y. Liu D. Wang Y. Zhang F. Xu Y. Pu Q. (2023). Combined analysis of the proteome and metabolome provides insight into microRNA-1174 function in *Aedes aegypti* mosquitoes. Parasit. Vectors 16 (1), 271. Published 2023 Aug 9. 10.1186/s13071-023-05859-1 37559132 PMC10413549

[B22] Maduwage K. Isbister G. K. (2014). Current treatment for venom-induced consumption coagulopathy resulting from snakebite. PLoS Negl. Trop. Dis. 8 (10), e3220. Published 2014 Oct 23. 10.1371/journal.pntd.0003220 25340841 PMC4207661

[B23] Mahmoudi G. A. Ahadi M. Fouladvand A. Rezaei B. Bodagh Z. Astaraki P. Evaluation of allergic reactions following intravenous infusion of polyvalent antivenom in snakebite patients following intravenous infusion of polyvalent antivenom in snakebite patients. Antiinflamm. Antiallergy Agents Med. Chem. 2021;20(4):367–372. 10.2174/1871523020666210204143756 33563188

[B24] Matsumori R. Miyazaki T. Shimada K. Kume A. Kitamura Y. Oshida K. (2013). High levels of very long-chain saturated fatty acid in erythrocytes correlates with atherogenic lipoprotein profiles in subjects with metabolic syndrome. Diabetes Res. Clin. Pract. 99 (1), 12–18. 10.1016/j.diabres.2012.10.025 23146370

[B25] Mickiewicz B. Vogel H. J. Wong H. R. Winston B. W. (2013). Metabolomics as a novel approach for early diagnosis of pediatric septic shock and its mortality. Am. J. Respir. Crit. Care Med. 187 (9), 967–976. 10.1164/rccm.201209-1726OC 23471468 PMC3707368

[B26] Minghui R. Malecela M. N. Cooke E. Abela-Ridder B. (2019). WHO's Snakebite Envenoming Strategy for prevention and control. Lancet Glob. Health 7 (7), e837–e838. 10.1016/S2214-109X(19)30225-6 31129124

[B27] Mota S. M. B. Albuquerque PLMM Meneses G. C. da Silva Junior G. B. Martins A. M. C. De Francesco Daher E. (2021). Role of endothelial biomarkers in predicting acute kidney injury in Bothrops envenoming. Toxicol. Lett. 345, 61–66. 10.1016/j.toxlet.2021.04.010 33872748

[B28] Namba T. Huang X. L. Shu Y. Z. Huang S. L. Hattori M. Kakiuchi N. (1989). Chronotropic effect of the methanolic extracts of the plants of the Paris species and steroidal glycosides isolated from P. Vietnamensis on spontaneous beating of myocardial Cells1. Planta Med. 55 (6), 501–505. 10.1055/s-2006-962080 17262471

[B29] Ní R. S. Bender K. Lacey N. Brennan L. Powell F. C. (2012). The fatty acid profile of the skin surface lipid layer in papulopustular rosacea. Br. J. Dermatol. 166 (2), 279–287. 10.1111/j.1365-2133.2011.10662.x 21967555

[B30] Patil R. Aswar U. Vyas N. (2023). Pterostilbene ameliorates type-2 diabetes mellitus - induced depressive-like behavior by mitigating insulin resistance, inflammation and ameliorating HPA axis dysfunction in rat brain. Brain Res. 1817, 148494. 10.1016/j.brainres.2023.148494 37478963

[B31] Pietrafesa R. Siesto G. Tufariello M. Palombi L. Baiano A. Gerardi C. (2023). A multivariate approach to explore the volatolomic and sensory profiles of craft Italian Grape Ale beers produced with novel *Saccharomyces cerevisiae* strains. Front. Microbiol. 14, 1234884. Published 2023 Jul 27. 10.3389/fmicb.2023.1234884 37577427 PMC10414987

[B32] Qiu S. Cai Y. Yao H. Lin C. Xie Y. Tang S. (2023). Small molecule metabolites: discovery of biomarkers and therapeutic targets. Signal Transduct. Target Ther. 8 (1), 132. Published 2023 Mar 20. 10.1038/s41392-023-01399-3 36941259 PMC10026263

[B33] Ralph R. Faiz M. A. Sharma S. K. Ribeiro I. Chappuis F. (2022). Managing snakebite. BMJ 376, e057926. Published 2022 Jan 7. 10.1136/bmj-2020-057926 34996773 PMC9278408

[B34] Sanchez-Castro E. E. Pajuelo-Reyes C. Tejedo R. Soria-Juan B. Tapia-Limonchi R. Andreu E. (2021). Mesenchymal stromal cell-based therapies as promising treatments for muscle regeneration after snakebite envenoming. Front. Immunol. 11, 609961. Published 2021 Feb 3. 10.3389/fimmu.2020.609961 33633730 PMC7902043

[B35] Sebastin Santhosh M. Hemshekhar M. Thushara R. M. Devaraja S. Kemparaju K. Girish K. S. (2013). Vipera russelli venom-induced oxidative stress and hematological alterations: amelioration by crocin a dietary colorant. Cell. Biochem. Funct. 31 (1), 41–50. 10.1002/cbf.2858 22893269

[B36] Sissler M. (2021). Decoding the impact of disease-causing mutations in an essential aminoacyl-tRNA synthetase. J. Biol. Chem. 297 (6), 101386. 10.1016/j.jbc.2021.101386 34752820 PMC8626572

[B37] Stifel U. Wolfschmitt E. M. Vogt J. Wachter U. Vettorazzi S. Tews D. (2022). Glucocorticoids coordinate macrophage metabolism through the regulation of the tricarboxylic acid cycle. Mol. Metab. 57, 101424. 10.1016/j.molmet.2021.101424 34954109 PMC8783148

[B38] Suhita R. Begum I. Rashid M. Chandran V. P. Shastri S. A. Kantamneni R. (2022). Systematic review and meta-analysis of global prevalence of neurotoxic and hemotoxic snakebite envenomation. East Mediterr. Health J. 28 (12), 909–916. Published 2022 Dec 21. 10.26719/emhj.22.090 36573572

[B39] Sun R. Li Y. Cai M. Cao Y. Piao X. (2019). Discovery of a new biomarker pattern for differential diagnosis of AcuteIschemic stroke using targeted metabolomics. Front. Neurol. 10, 1011. Published 2019 Sep 19. 10.3389/fneur.2019.01011 31608005 PMC6761218

[B40] Trevett A. J. Lalloo D. G. Nwokolo N. C. Naraqi S. Kevau I. H. Theakston R. D. (1995). The efficacy of antivenom in the treatment of bites by the Papuan taipan (Oxyuranus scutellatus canni). Trans. R. Soc. Trop. Med. Hyg. 89 (3), 322–325. 10.1016/0035-9203(95)90562-6 7660450

[B41] Udvardy A. Szolnoki C. T. Gombos R. Papp G. Kováts É. Joó F. (2020). Mechanochemical P-derivatization of 1,3,5-triaza-7-phosphaadamantane (PTA) and silver-based coordination polymers obtained from the resulting phosphabetaines. Molecules 25 (22), 5352. Published 2020 Nov 16. 10.3390/molecules25225352 33207789 PMC7697749

[B42] Uhrig M. L. Couto A. S. Alves M. J. Colli W. de Lederkremer R. M. (1997). Trypanosoma cruzi: nitrogenous-base-containing phosphatides in trypomastigote forms--isolation and chemical analysis. Exp. Parasitol. 87 (1), 8–19. 10.1006/expr.1997.4181 9287953

[B43] Wang T. Liu J. Luo X. Hu L. Lu H. (2021). Functional metabolomics innovates therapeutic discovery of traditional Chinese medicine derived functional compounds. Pharmacol. Ther. 224, 107824. 10.1016/j.pharmthera.2021.107824 33667524

[B44] Wei H. Yin Y. Yang W. Zhu J. Chen L. Guo R. (2023). Nuciferine induces autophagy to relieve vascular cell adhesion molecule 1 activation via repressing the Akt/mTOR/AP1 signal pathway in the vascular endothelium. Front. Pharmacol. 14, 1264324. Published 2023 Sep 28. 10.3389/fphar.2023.1264324 37841916 PMC10569124

[B45] Wu X. Ma G. L. Chen H. W. Zhao Z. Y. Zhu Z. P. Xiong J. (2023). Antibacterial and antibiofilm efficacy of the preferred fractions and compounds from Euphorbia humifusa (herba euphorbiae humifusae) against *Staphylococcus aureus* . J. Ethnopharmacol. 306, 116177. 10.1016/j.jep.2023.116177 36681167

[B46] Yanagisawa N. Shimada K. Miyazaki T. Kume A. Kitamura Y. Sumiyoshi K. (2008). Enhanced production of nitric oxide, reactive oxygen species, and pro-inflammatory cytokines in very long chain saturated fatty acid-accumulated macrophages. Lipids Health Dis. 7, 48. Published 2008 Nov 28. 10.1186/1476-511X-7-48 19038055 PMC2613382

[B47] Ye J. Qian W. Chen N. Hu Z. Ye S. The clinical efficacy of Zuqing Xu "Wuduling" powder for snake injury on the swelling of the affected limb bitten by Agkistrodon *halys* of the affected limb bitten by Agkistrodon halys. Biotechnol. Genet. Eng. Rev. 22, 1, 18, . 2023. 10.1080/02648725.2023.2191085 36946536

[B48] Zeng B. Wei A. Zhou Q. Yuan M. Lei K. Liu Y. (2022). Andrographolide: a review of its pharmacology, pharmacokinetics, toxicity and clinical trials and pharmaceutical researches. Phytother. Res. 36 (1), 336–364. 10.1002/ptr.7324 34818697

[B49] Zhou J. Sun F. Zhang W. Feng Z. Yang Y. Mei Z. (2024). Novel insight into the therapeutical potential of flavonoids from traditional Chinese medicine against cerebral ischemia/reperfusion injury. Front. Pharmacol. 15, 1352760. Published 2024 Feb 29. 10.3389/fphar.2024.1352760 38487170 PMC10937431

