# Supplementary material for: Metabolomic analysis of Agkistrodon haly venom poisoning mouse treatment by Jidesheng snake pill based on GC-MS
Source: Front Pharmacol. 2024 Aug 1;15:1419609. doi: 10.3389/fphar.2024.1419609 (PMC11324443; doi:10.3389/fphar.2024.1419609)
Supplement: Supplementary file 1 [file DataSheet1.PDF]

Supplementary Table 1. The gradient elution condition

Time/Min	A（%）	B（%）
Initial	95	5
3	75	25
8.5	55	45
14	5	95
17	2	98
17.2	95	5
20	95	5

Supplementary Table 2. Identification of chemical constituents from JDS by UHPLCMS/MS

NO.	Compound Name	Molecular formula	Pub CHEM	RT min	Mass error (ppm)	Ionization model
1	Bufotenine	C12H16N2O	10257	2.15	0.5	[M+H]+
2	Levoglucosan	C6H10O5	2724705	2.27	1.2	[M+H-H2O]+
3	5-Ureidovaleric acid	C6H12N2O3	458589	2.47	0.2	[M+H-CH3NO2]+
4	Pyrogallol	C6H6O3	1057	2.58	1	[M+H]+
5	4-Sulfocatechol	C14H29NO2	8899	3.25	1.7	[M+H]+
6	Esmolol	C16H25NO4	59768	3.38	1.8	[M+H-H2O]+
7	4H-1-Benzopyran-4-one, 6-arabinopyranosyl-8-.beta.-D-glucopyranosyl-5,7-dihydroxy-2-(4-hydroxyphenyl)-	C26H28O14	13644660	4.18	1.4	[M+H]+
8	4-Methyl-2-oxo-1(2H)-quinolineacetic acid	C12H11NO3	268297	4.56	1.9	[M+H]+
9	Nicotiflorin	C27H30O15	5318767	4.7	1.6	[M+H]+
10	Jasminoside R	C22H34O12	45783213	4.76	13.7	[M+NH4]+
11	Isovitexin	C21H20O10	162350	4.84	1.3	[M+H]+
12	Spinosine	C28H32O15	155692	4.97	1.6	[M+H-C6H10O5]+
13	2,3,14,20,23,25-Hexahydroxycholest-7-en-6-one	C27H44O7	23900102	5.09	1.2	[M+H-2H2O]+
14	Diosmin	C28H32O15	5281613	5.45	1.4	[M+H]+
15	Astragalin	C21H20O11	5282102	5.64	1.9	[M+H]+
16	Isofraxidin	C11H10O5	5318565	6.3	1.4	[M+H]+
17	5-Hydroxy-3-(4-methoxyphenyl)-4-oxo-4H-chromen-7-yl	C28H32O14	45782929	7.02	1.3	[M+H]+

	6-O-(6-deoxyhexopyranosyl)hexopyranoside					
18	Gabapentin related compound D	C18H29NO3	29980616	7.14	1.8	[M+H]+
19	Luteolin	C15H10O6	5280445	7.65	1.9	[M+H]+
20	Apiin	C26H28O14	5280746	8.85	2.1	[M+H-C11H18O9]+
21	5,8-Dihydroxy-2-(4-methoxyphenyl)-4-oxo-4H-chromen-7-yl 2-O-(6-O-acetyl-.beta.-D-allopyranosyl)-.beta.-D-glucopyranoside	C30H34O17	21576582	9.01	2.5	[M+H-C14H22O11]+
22	Limettin	C11H10O4	2775	9.35	1.4	[M+H]+
23	Undecanedioic acid	C11H20O4	15816	10.08	1.9	[M+H-2H2O]+
24	4-Acetoxy-8-(3-keto-2-pent-2-enylcyclopenten-1-yl)caprylic acid	C20H30O5	57481829	11.02	2.2	[M+H-C2H4O2]+
25	Cinobufagin	C26H34O6	11969542	11.4	1.9	[M+H]+
26	7.alpha.-Hydroxy-3-oxo-4-cholestenoic acid	C27H42O4	91746147	11.78	1.9	[M+H-H2O]+
27	Asperglaucide	C27H28N2O4	124319	12	0.5	[M+H]+
28	Nitogenin	C27H42O3	99474	13.28	1.2	[M+H]+
29	9-Oxo-10(E),12(E)-octadecadienoic acid	C18H30O3	5283011	13.66	1.9	[M+H-H2O]+
30	Gallic acid	C7H6O5	370	2.14	1.3	[M-H]-
31	4-Chloro-8-methoxy-5H-pyrimido[5,4b] indole	C11H8ClN3O	5399944	2.64	0.3	[M-H]-
32	2-[3-oxo-2-(5-sulfooxypent-2-enyl)cyclopentyl]acetic acid	C12H18O7S	75075824	4.18	4.4	[M-H]-
33	2-Chloro-N-(2-hydroxyphenyl)nicotinamide	C12H9ClN2O2	1211386	4.39	13.8	[M-H]-
34	Ellagic acid	C14H6O8	5281855	4.94	0.6	[M-H]-
35	Phenol glucuronide	C12H14O7	87235	5.77	0.3	[M-H-H2O]-
36	1,8-Dihydroxy-9-ox	C13H10O6	45782742	6.15	0.8	[M-H]-

	o-2,3-dihydro-1H-cyclopenta[b]chromene-6-carboxylic acid					
37	Azelaic acid	C9H16O4	2266	6.35	0.7	[M-H]-
38	JWH 250 5-hydroxyindole metabolite	C22H25NO3	91736661	6.68	2.1	[M-H-C5H11]-
19	Luteolin	C15H10O6	5280445	7.57	0.5	[M-H]-
39	Ethyl 3-hydroxybenzoate	C9H10O3	24522	8.23	0.7	[M-H]-
40	7,8,4'-Trihydroxyflavone	C15H10O5	688853	8.75	0.1	[M-H]-
41	Diosmetin	C16H12O6	5281612	8.92	0.4	[M-H]-
42	(11E,15Z)-9,10,13-Trihydroxy-11,15-octadecadienoic acid	C18H32O5	23872026	9.34	0.6	[M-H]-
43	9-Octadecenoic acid, 5,8,11-trihydroxy-	C18H34O5	24096399	10.16	0.7	[M-H]-
44	(Z)-9,10,11-Trihydroxy-12-octadecenoic acid	C18H34O5	75536014	11.1	0.5	[M-H]-
45	Pennogenin-3-O-tetraglycoside	C51H82O21	24121280	11.65	1.8	[M+HCOO]-
46	Spirostane + 2O, -2H, O-Hex-dHex-dHex	C45H72O18	139291973	11.74	0.6	[M+HCOO]-
47	Furostane base -1H2O -2H + 1O, O-Hex, O-Pen-dHex	C44H70O17	139291897	11.83	1.1	[M+HCOO]-
48	(9Z,12E)-15,16-Dihydroxyoctadeca-9,12-dienoic acid	C18H32O4	45783236	12.02	0.8	[M-H]-
49	[3-Me-His2] TRH	C17H24N6O4	3548445	12.13	15.5	[M-H]-
50	Polyphyllin vi	C39H62O13	71307571	12.52	1.2	[M-H]-
51	9,10-DiHOME	C18H34O4	25320858	12.61	0.4	[M-H]-
52	Peucenin	C15H16O4	68477	12.7	12.4	[M-H]-
53	Polyphyllin e	C51H82O20	102594501	13.05	0.1	[M-H]-
54	Dioscin	C45H72O16	119245	13.2	2.7	[M+HCO2]-
55	Polyphyllin i(chonglou saponin i)	C44H70O16	71571451	13.31	2.8	[M+HCO2]-
56	12(13)-EpOME	C18H32O3	5356421	13.52	1.5	[M-H]-

57	9-HpODE	C18H32O4	6439847	13.65	0.7	[M-H-H2O]-
58	6-Hydroxyoctadec-4-enoic acid	C18H34O3	51136456	14	0.8	[M-H]-

Supplementary table 3. Survival of mice at 7 days after exposure to different doses of venom

dose mg/kg	total	death
1	6	0
2	6	1
4	6	2
6	6	3
8	6	6
10	6	6

Supplementary table 4. Death and mortality of mice in different dose groups within 7 days

Motality	Concentration （mg/kg） Estimated value	95%CIs Lower	Upper	Motality	Concentration （mg/kg） Estimated value	95%CIs Lower	Upper
0.01	0.073	-5.52	1.968	0.55	5.243	3.987	6.907
0.02	0.648	-4.325	2.379	0.6	5.513	4.298	7.348
0.03	1.013	-3.571	2.644	0.65	5.791	4.596	7.827
0.04	1.287	-3.007	2.846	0.7	6.084	4.889	8.354
0.05	1.51	-2.551	3.014	0.75	6.401	5.184	8.943
0.06	1.7	-2.164	3.158	0.8	6.753	5.493	9.618
0.07	1.867	-1.827	3.286	0.85	7.164	5.834	10.425
0.08	2.016	-1.527	3.403	0.9	7.68	6.242	11.462
0.09	2.151	-1.255	3.51	0.91	7.805	6.337	11.715
0.1	2.276	-1.007	3.61	0.92	7.941	6.44	11.991
0.15	2.793	0.006	4.041	0.93	8.09	6.552	12.295
0.2	3.204	0.786	4.409	0.94	8.257	6.676	12.637
0.25	3.556	1.431	4.749	0.95	8.447	6.816	13.027
0.3	3.873	1.984	5.079	0.96	8.67	6.979	13.488
0.35	4.166	2.471	5.412	0.97	8.944	7.177	14.057
0.4	4.444	2.905	5.756	0.98	9.309	7.437	14.816
0.45	4.713	3.297	6.116	0.99	9.883	7.841	16.017

Supplementary table 5. The quantitative real-time polymerase chain reaction primer sequences.

Species	Genes	Forward primer	Reverse primer
Mouse	TNF-α	TACTGAACTTCGGGGTGATTGGTCC	CAGCCTTGTCCCTTGAAGAGAACC
Mouse	GAPDH	AGGCCGGTGCTGAGTATGTC	TGCCTGCTTCACCACCTTCT

Supplementary table 6. Changes of muscle metabolites in mice. (Ah vemon groups vs. control group)

metabolite	logFC			P.Value
	4h vs.before	24h vs.before	7d vs.before	

L-Threonine	-2.103	-0.266	0.424	＜0.001
Tryptophan	-2.108	-0.291	0.403	＜0.001
phenylalanine	-2.007	-0.341	0.583	＜0.001
serine	-2.023	-0.483	0.553	＜0.001
isoleucine	-2.100	-0.401	0.435	＜0.001
Succinic acid	2.378	0.174	0.377	＜0.001
Valine	-1.998	-0.506	0.579	＜0.001
proline	-1.981	-0.299	0.593	＜0.001
Pentadecane	-1.849	-0.668	0.729	＜0.001
glutamic acid	-1.658	-0.289	0.985	＜0.001
Threonine	-2.205	-0.397	0.188	＜0.001
lysine	-2.238	-0.758	0.136	＜0.001
methionine	-2.031	-0.465	0.481	＜0.001
2-Aminoadipic acid	-1.896	-0.369	0.678	＜0.001
1-Aminocyclopropane-1-carboxylic acid	-2.025	-0.529	0.493	＜0.001
Glutathione	-1.751	-0.698	0.794	＜0.001
Creatinine	-1.992	-0.369	0.523	＜0.001
Tyrosine	-2.235	-0.509	0.115	＜0.001
glycine	-1.854	-0.968	0.610	＜0.001
Ornithine	-1.608	0.370	0.868	＜0.001
aspartic acid	-1.577	-0.011	0.996	＜0.001
d-Prolyl-d-proline	-2.464	-0.874	-0.471	＜0.001
L-Serine,	-2.150	0.077	-0.184	＜0.001
alanine	-2.019	-0.549	0.390	＜0.001
Histidine	-2.231	-0.652	0.012	＜0.001
Alanine, N-methyl-N	-1.733	-0.576	0.774	＜0.001
leucine	-2.039	-0.284	0.314	＜0.001
2-n-Hexylphenol	-2.235	-0.696	-0.177	＜0.001
Lactic acid	-2.246	-1.252	-0.278	＜0.001
a-Ketoglutaric aicd	-2.249	-0.226	-0.659	＜0.001
DHA	1.516	1.863	-0.013	＜0.001
Sarcosine	-1.821	-2.018	-0.459	＜0.001
Gamma-Aminobutyric acid	-1.733	-0.745	0.584	＜0.001
Citric acid	-1.059	-0.085	1.299	＜0.001
Myristic acid	-0.926	-0.113	1.403	＜0.001
Cyclotetrasiloxane, octamethyl-	1.305	0.392	-0.889	＜0.001
L-alpha-Aminobutyric acid	-1.333	0.939	0.139	＜0.001
Hydroxybenzoic acid	0.154	-0.108	1.886	＜0.001
Levulinic acid	0.674	-1.067	1.062	＜0.001
Malic acid	-1.131	0.019	1.134	＜0.001
2-Hydroxyisobutyric acid	1.044	2.297	1.155	＜0.001
Isocitric acid	-1.028	-0.644	1.111	＜0.001
Undecanoic acid	-0.625	0.221	1.450	＜0.001

Trichloroethanol	-1.808	-0.768	-0.035	＜0.001
Pyroglutamic acid	-1.506	-0.365	0.669	＜0.001
Itaconic acid	0.470	1.562	1.925	＜0.001
Oleic acid	-0.587	1.228	1.093	＜0.001
Heptadecane	-1.700	-0.581	0.198	＜0.001
Linoleic acid	-0.650	1.171	1.038	＜0.001
Linolelaidic acid	-0.618	1.175	1.041	＜0.001
Heneicosanoic acid	-0.721	0.048	1.109	＜0.001
Oxalic acid	-0.251	0.283	1.665	＜0.001
cis-Aconitic acid	-0.449	0.395	1.509	0.001
trans-Vaccenic acid	0.173	1.231	1.717	0.001
beta-Alanine	-1.822	-0.937	-0.386	0.001
Malonic acid	1.567	-0.195	0.647	0.002
Fumaric acid	-0.531	0.184	1.350	0.002
Citraconic acid	1.739	0.776	1.566	0.002
Eicosatrienoic acid	0.752	1.869	1.051	0.002
Glyoxylic acid	1.660	0.217	1.165	0.003
Isobutyl methyl carbonic acid	1.064	0.238	-0.732	0.003
Tridecanoic acid	-0.750	-0.727	0.787	0.003
Dodecane	-1.015	-0.881	0.601	0.004
Cabamic acid	1.307	-0.419	-0.054	0.005
Pentadecanoic acid	0.701	0.700	1.730	0.006
Eicosadienoic acid	0.233	1.234	1.368	0.006
Tricosanoic acid	1.534	1.450	0.605	0.007
gamma-Linolenic acid	-1.037	-0.316	0.633	0.009
10-Heptadecenoic acid	-0.148	0.188	1.282	0.015
11-Eicosenoic acid	-0.485	1.101	0.011	0.016
2-Hydroxybutyric acid	0.254	1.169	-0.478	0.017
13, 16-Docosadienoic acid	0.842	1.385	1.335	0.019
Palmitelaidic acid	0.006	0.245	1.406	0.020
Hexanoic acid	1.433	0.287	0.310	0.025
Cyclopentasiloxane, decamethyl-	1.284	0.661	-0.091	0.031
Benzene,propyl-	-1.037	0.264	0.367	0.034
Caprinoic acid	0.102	-0.090	1.281	0.034
Tetrachlorethane	-0.106	0.915	-0.627	0.035
Hexachlorethane	-1.013	0.293	-0.628	0.046
Decanoic acid	0.446	1.077	1.072	0.049

Supplementary table 7. Changes of muscle metabolites in mice. (Ah vemon+JDS groups vs. Ah vemon groups)

metabolite	logFC	t	P.Value
Adipic acid	0.857	2.658	0.014
Tridecanoic acid	0.692	2.364	0.027
Heneicosanoic acid	-0.956	-3.870	＜0.001
Lignoceric acid	-1.131	-3.834	＜0.001

Behenic acid	-1.127	-3.813	＜0.001
d4-Alanine	-1.124	-3.749	0.001
Trichloroethanol	-0.823	-3.639	0.001
Arachidic acid	-1.089	-3.637	0.001
Decanoic acid	-1.075	-3.591	0.002
Undecanoic acid	-0.737	-3.222	0.004
Cyclotetrasiloxane, octamethyl-	-0.635	-2.874	0.009
Erucic acid	-0.952	-2.681	0.014
Adipic acid	0.857	2.658	0.014
11,14-Eicosadienoic acid	-0.736	-2.459	0.022
Phthalic acid	-0.802	-2.458	0.022
Xylene	-0.880	-2.417	0.024
Nicotinamide	-0.822	-2.402	0.025
10-Heptadecenoic acid	-0.743	-2.384	0.026
DBP	-0.849	-2.367	0.027
Tridecanoic acid	0.692	2.364	0.027
DHA	-0.449	-2.318	0.030
Docosadienoic acid	-0.709	-2.237	0.036
Isobutyl methyl carbonic acid	-0.628	-2.128	0.045

Supplementary Figure 1. The positive ion mode of total ion chromatogram of JDS obtained by UHPLC-MS/MS analysis.

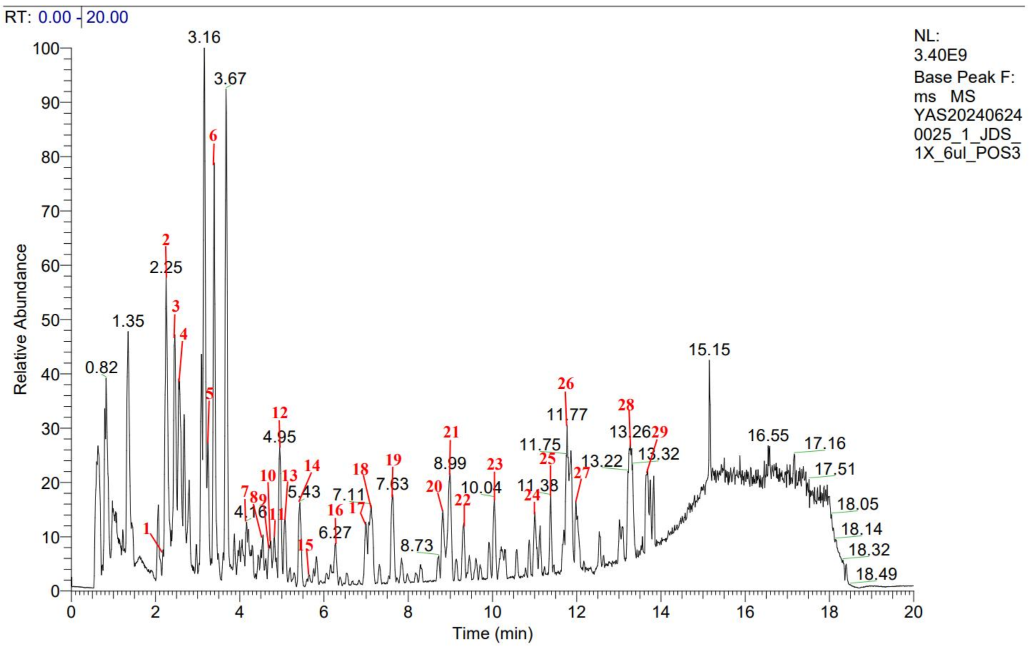

Supplementary Figure 2. The negative ion mode of total ion chromatogram of JDS obtained by UHPLC-MS/MS analysis.

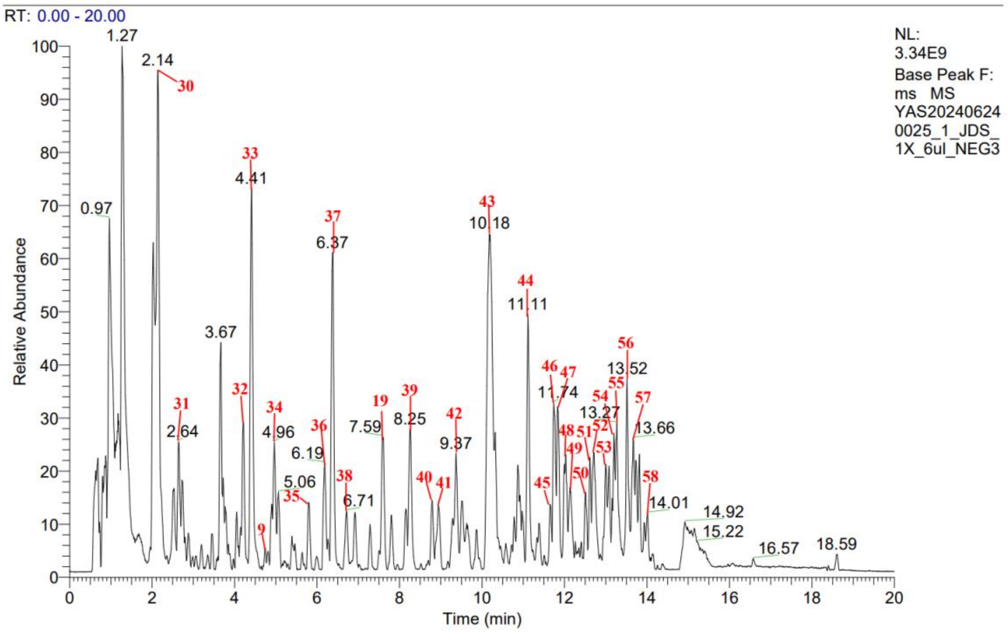
RT: 0.00 - 20.00
NL:
3.34E9
Base Peak F:
ms MS
YAS20240624
0025_1_JDS_
1X_6ul_NEG3
Relative Abundance
Time (min)
0.97
1.27
2.14
30
31
2.64
3.67
32
33
4.41
34
4.96
9
5.06
35
36
6.19
37
6.37
38
6.71
7.59
19
39
8.25
40
41
42
9.37
43
10.18
44
11.11
45
46
47
11.74
48
49
50
51
52
53
54
55
13.27
56
13.52
57
13.66
58
14.01
14.92
15.22
16.57
18.59
